# Selecting and implementing overview methods: implications from five exemplar overviews

**DOI:** 10.1186/s13643-017-0534-3

**Published:** 2017-07-18

**Authors:** Alex Pollock, Pauline Campbell, Ginny Brunton, Harriet Hunt, Lise Estcourt

**Affiliations:** 1 Nursing Midwifery and Allied Health Professions (NMAHP) Research Unit, Glasgow Caledonian University, Research Unit, 6th Floor Govan Mbeki Building, Cowcaddens Road, Glasgow, G4 0BA UK; 2 UCL Institute of Education, University College London, 20 Bedford Way, London, WC1H 0AL UK; 3 University of Exeter Medical School, St Luke’s campus, Exeter, Devon EX1 1TE UK; 4 NHS Blood and Transplant Oxford and Radcliffe Department of Medicine, University of Oxford, Level 2, John Radcliffe Hospital, Oxford, OX3 9BQ UK

**Keywords:** Challenges, Methods, Overviews, Quality assessment, Synthesis

## Abstract

**Background:**

Overviews of systematic reviews are an increasingly popular method of evidence synthesis; there is a lack of clear guidance for completing overviews and a number of methodological challenges. At the UK Cochrane Symposium 2016, methodological challenges of five overviews were explored. Using data from these five overviews, practical implications to support methodological decision making of authors writing protocols for future overviews are proposed.

**Methods:**

Methods, and their justification, from the five exemplar overviews were tabulated and compared with areas of debate identified within current literature. Key methodological challenges and implications for development of overview protocols were generated and synthesised into a list, discussed and refined until there was consensus.

**Results:**

Methodological features of three Cochrane overviews, one overview of diagnostic test accuracy and one mixed methods overview have been summarised. Methods of selection of reviews and data extraction were similar. Either the AMSTAR or ROBIS tool was used to assess quality of included reviews. The GRADE approach was most commonly used to assess quality of evidence within the reviews.

Eight key methodological challenges were identified from the exemplar overviews. There was good agreement between our findings and emerging areas of debate within a recent published synthesis. Implications for development of protocols for future overviews were identified.

**Conclusions:**

Overviews are a relatively new methodological innovation, and there are currently substantial variations in the methodological approaches used within different overviews. There are considerable methodological challenges for which optimal solutions are not necessarily yet known. Lessons learnt from five exemplar overviews highlight a number of methodological decisions which may be beneficial to consider during the development of an overview protocol.

**Electronic supplementary material:**

The online version of this article (doi:10.1186/s13643-017-0534-3) contains supplementary material, which is available to authorized users.

## Background

Overviews of systematic reviews are an increasingly popular method of evidence synthesis [1, 2], and there are a growing number of resources, including guidelines, recommendations, descriptions and systematic reviews, relating to overview methods [2–6]. While there are some areas of agreement in relation to optimal overview methods, particularly in the early stages of overview completion [5], there remains considerable uncertainty around some key areas of methodology [3, 5, 7, 8] and a need for clearer standards and reporting guidance, supported by research evidence, to enhance methodological quality of overviews [1–3, 5, 6].

At the UK Cochrane Symposium in 2016, a workshop focusing on the methods and challenges associated with overviews included presentations relating to five selected ongoing or recently completed overviews [9]. These were selected in order to highlight practical variations in the methods adopted within different overviews and to provide tangible examples of the decisions and challenges associated with the completion of an overview. During and following this workshop, we explored practical issues associated with planning and preparing these exemplar overviews, discussed the impact of methodological decisions and reached consensus on key methodological challenges and potential implications for future overview authors.

Subsequent to our author group reaching consensus, Ballard [3] published the results of a scoping review of methodological guidance for overviews. This synthesis identified areas where there was consensus relating to overview methods and highlighted five key areas where there was debate or uncertainty including “(i) overlapping systematic reviews, (ii) the scope of systematic reviews, (iii) evaluating the quality and reporting of included research, (iv) updating included systematic reviews, and (v) synthesizing and reporting the results of included systematic reviews” [3]. Since the search period of this comprehensive synthesis, a number of further papers relevant to overview methods have been published adding to the discussion and debate around key areas of uncertainty. These include summaries of guidance relating to overview methods [5], descriptions and debate relating to the use of GRADE [10–12] and AMSTAR [13], the development of a new tool to assess risk of bias (ROBIS [14]), and protocols for ongoing work in this field [15, 16]. The specific debate around methodological challenges presented by Ballard [3], and these subsequent related publications, afforded us a timely opportunity to compare the results of our independent consensus arising from our five exemplar overviews and to explore the practical implications of the current uncertainties around overview methods for authors planning protocols for new overviews.

Our aim is therefore to use five exemplar overviews:

(i) To provide practical examples of methodological approaches to the planning and preparing of overviews and discuss the impact of methodological decisions
(ii) To explore methodological challenges reported by overview authors and compare these with areas of debate identified within current literature
(iii) To discuss practical implications which may support methodological decision making during development of protocols for future overviews

## Methods

### Methodological features of exemplar overviews

Methods of the five exemplar overviews were tabulated, relating to all key stages in the process of completing an overview. Justification for the selection and use of the methods at each stage were provided by the overview authors, based on the presentations provided at the Cochrane Workshop and supplemented by discussion (authors representing all five exemplar overviews are included as authors on this paper). Points of agreement and dissonance were highlighted and discussed. The key methodological challenges identified by each overview author were discussed at the workshop and synthesised into a list, with similar challenges merged. A description of each challenge, and the solutions (if any) implemented within individual overviews, was developed through discussion. A list of potential implications for future overview authors, arising from the practical experiences within these exemplar overviews, was developed iteratively. This initially comprised a list of key methodological decisions made by the overview authors arising from the tabulated descriptions of methods and methodological challenges; this was circulated amongst the overview authors who added to, and refined until consensus was reached on the final list.

### Key challenges within exemplar overviews and complementarity with published literature on overview methods

The key methodological challenges identified within each of the exemplar overviews were systematically compared and contrasted with the recognised areas of debate [3], which were published after consensus had been reached on the methodological challenges identified within our exemplar overviews. Each of the methodological challenges from our exemplar overviews were tabulated, and overview authors considered levels of complementarity [17, 18] between the recognised areas of debate and the methodological challenges identified from our exemplar overviews, applying categories of “agreement” (the methodological challenges identified within the exemplar overviews is in agreement with the findings of Ballard [3]), “dissonance” (the methodological challenges identified within the exemplar overviews differs from, or conflicts with, the findings of Ballard [3]) or “silence” (the methodological challenges identified within the exemplar overviews were not addressed within the findings of Ballard [3, 17, 18]. One author applied the initial categorisation, which were then appraised by the other authors and any areas of disagreement highlighted. Disagreements were discussed between all authors until consensus was reached, the findings of other recent publications were considered, and any changes from the original categorisation noted.

### Implications for development of protocols for future overviews

Finally, based on the presentations on the methods of the exemplar overviews from the Cochrane Symposium which all provided a chronological description of the overview process and the synthesis of methodological features, common features and differences between our exemplar overviews were identified and agreed. Considering these features and differences alongside the perceived methodological challenges and complementarity with published literature, overview authors then debated and agreed key implications for the development of protocols for future overviews.

## Results

### Methodological features of exemplar overviews

Table 1 summarises the key methodological features of the five exemplar overviews. Three of the overviews are Cochrane overviews [19–21], one is an overview of reviews of diagnostic test accuracy (DTA) [22], and one a mixed method overview carried out by the EPPI centre [23]. Two of the Cochrane overviews are produced by the same research team (AP is an author on [19] and [21]), while the remaining three are each produced by distinct author groups. Detailed protocols describing the planned methods were agreed and made available a priori for all five of the overviews; two of the overviews are now complete and published [19, 23], while three are in the final stages of completion and write-up [20–22]. Justification for the selection and use of the methods as described in the protocol and used at each stage of the overview process are provided in Table 1. Characteristics of the included reviews and any data relating to the results of meta-analyses which were extracted and reported within the systematic reviews are summarised in Table 2.

**Table 1:** Methodological features of exemplar overviews

	Pollock [19]	McClurg [21]	Estcourt [20]	Hunt [22]	Brunton [23]
Type of overview	Cochrane overview of reviews	Cochrane overview of reviews	Cochrane overview of reviews	Overview of reviews of diagnostic test accuracy	Mixed method overview of reviews
Aim/question of overview	To synthesise systematic reviews of interventions to improve upper limb function after stroke	To synthesise Cochrane reviews of conservative interventions for the prevention or treatment of female urinary incontinence	To summarise the evidence in Cochrane reviews of the effectiveness and safety of red cell transfusions for treatment or prevention of complications experienced by people with sickle cell disease	To summarise evidence from studies of the accuracy of diagnostic tests of brief cognitive assessments for identifying dementia in primary care	Overview of workplace health promotion interventions
Inclusion criteria: participants, interventions, outcomes	Participants: stroke	Participants: female adults with urinary incontinence	Participants: people with sickle cell disease	Participants: all adults aged 18 years or over recruited from a primary care or general practice population	Participants: any
					Interventions: any interventions delivered in a workplace setting
	Interventions: any	Interventions: any conservative intervention (definition provided)	Interventions: red cells transfusions		
				Index test: brief cognitive assessments	
	Outcomes: upper limb function, impairment or activity of daily living	Outcomes: any	Outcomes: mortality, sickle-cell related complications, adverse events, quality of life, red cell transfusion requirements, and hospital admissions	Outcomes: diagnostic test result (positive/negative)	Outcomes: healthcare or wellbeing outcomes
				Other: diagnostic test accuracy information, other reported test outcomes	
Inclusion criteria: type of study	Systematic reviews including randomised controlled trials	Cochrane systematic reviews	Cochrane systematic reviews	Systematic reviews of diagnostic test accuracy	Systematic reviews in which the search strategy, inclusion criteria and quality assessment methods are described
					Published from 1995 to present; in English;
Search strategy	CDSR, DARE, PROSPERO	CDSR	CDSR	CDSR, EMBASE, MEDLINE, PsycInfo	MEDLINE, DARE, Cochrane Library, PsycInfo, DOPHER, ASSIA, ABI Inform, Scopus, Business Source Premier
	Reported line by line for CDSR and DARE	Reported line by line	Reported line by line	Pubmed search reported line by line	
					Details of the search strategy are available from the reviewers
Updating searches of out of date reviews	–	–	Searches of some included reviews were updated, in order to identify additional primary research studies, and update the original review to include these. The authors determined which reviews to update by using a process of judgement which systematically considered the search dates, presence of included or ongoing trials and relevance of the review topic	–	–
Selection of reviews	Two authors independently apply inclusion criteria	Two authors independently apply inclusion criteria	Two authors independently apply inclusion criteria	Two authors independently apply inclusion criteria	Two reviewers independently apply inclusion criteria
Data extraction	Characteristics of reviews; results of meta-analyses	Characteristics of reviews; results of meta-analyses	Characteristics of reviews; results of meta-analyses	Characteristics of reviews; results of meta analyses; diagnostic test accuracy information	Characteristics of reviews; results of meta-analyses; data from process evaluations
Assessment of quality of reviews	Modified AMSTAR: each of the original 11 AMSTAR questions/criteria were broken down into a series of dichotomous questions, and the answers (yes, no or unclear) to these dichotomous questions were used to determine the answer to the original AMSTAR questions	ROBIS	AMSTAR	AMSTAR and ROBIS	AMSTAR
			(Additional comments were added to each of the AMSTAR criteria to clarify the decision making process the authors had made)	(Note: employed both tools as there was an absence of guidance relating to the optimal tool for assessing reviews of DTA and these were the available options. Both tools were reported to provide comparable assessment results, but the ROBIS tool was considered to be easier to apply, and arguably more relevant, as this tool could be specifically tailored to reviews of DTA)	(Note: the AMSTAR scores were categorised into low (score 0–3), medium (score 4–7) or high (8–11))
	(Note: this assessment was completed prior to publication and availability of the ROBIS tool)				
Assessment of quality of evidence within reviews	GRADE approach, with use of algorithm (details presented and discussed elsewhere [10, 37])	GRADE approach, with use of algorithm, supplemented with input from key stakeholders (clinicians) and statisticians	GRADE approach, applied based on assessments provided in the included reviews, with two independent overview authors applying GRADE levels of evidence and any differences resolved through discussion or third party adjudication	No quality assessment was undertaken. Quality assessment measures from included systematic reviews reported narratively. Results showed that 2/13 reviews made no reference to quality assessment; 2/13 reviews referred to STARD criteria [38] as quality assessment; 2/13 did not report any details on methods or tool used; 6/13 used QUADAS-2 [39] or QUADAS; 1/13 review used a series of tools, including the United States Preventative Services Task Force’s design-specific quality criteria [40], National Institute for Health and Care Excellence (previously National Institute for Health and Clinical Excellence) methodology checklist [41], AMSTAR [42] and the Newcastle-Ottowa Scale for observational studies [43]	No quality assessment was undertaken: the objective of this overview was to provide a ‘systematic map’ of relevant reviews which aimed to identify characteristics associated with effectiveness
Data synthesis	Grouped according to GRADE, for intervention versus control, for each outcome explored. Highlighting evidence of beneficial effect, harm or no effect	Data grouped and presented according to type of urinary incontinence. Tabulated summary of systematic review evidence relating to all conservative interventions for urinary incontinence, signposting which systematic reviews address which interventions, with summary of details of the population of participants, comparisons, volume and quality of evidence	Reported using the GRADE approach, with presentation of statistical outcome data as determined by data available in the reviews	Synthesis of results across included studies using sensitivity and specificity measures, presented narratively and within tables	Framework synthesis to synthesise evidence from systematic reviews with other types of evidence (research of stakeholders’ experiences and perspectives; key policy documents). Data relating to moderator analyses to identify ‘successful’ intervention characteristics were tabulated
Statistical analysis	No statistical analysis was planned or completed	1. Network maps, created using Stata software, to illustrate the number of trial and participants within trials	No statistical analysis was planned or completed	No statistical analysis was completed	No statistical analysis was planned or completed
				(Note: additional analysis is ongoing relating to the assessment of test administration times, analysis of DTA across the most widely used brief cognitive assessments, and comparing diagnostic test performance across all systematic reviews)	
	A visual plot of the effect size relating to the effect of interventions on the primary outcome was produced (reproducing data from the included reviews)				
		2. Visual plot of effect size for comparisons of conservative intervention versus control, placebo or standard care for outcomes of condition-specific quality of life and symptomatic cure or improvement			
Summary of key findings	Two key summary tables were produced; each of these summarised the available evidence relating to 19 identified different interventions, highlighting the GRADE of the evidence, the direction of the effect (where GRADE was high or moderate), and key implications for clinical practice and research (see Additional file 1)	A ‘Summary of results’ table was planned, clearly indicating where there is evidence of an effect of conservative interventions, for each relevant intervention comparison and for both primary and secondary outcome of interest (see Additional file 2)	‘Overview of reviews’ table in a format similar to the ‘Summary of findings’ table as recommended in chapter 22 of the Cochrane Handbook for Systematic Reviews of Interventions [33]. These will include the primary outcomes of this overview for each intervention (see Additional file 2)	A summary of findings table was produced displaying review reference, component study reference, reference standard, index test, threshold, time taken, sample size and key demographic data, target condition, condition prevalence, accuracy data reported and notes. Additional summary tables were produced for comparisons at review level, non-DTA data reported, and individual brief cognitive assessments	Structured summary
					Executive summary
					Table of ‘key characteristics identified from effectiveness reviews, views studies and policy documents’
					A summary table in which information about each of the included reviews was provided, including the AMSTAR rating of the review. The direction of effect for different intervention types was also summarised, categorising evidence according to whether there was a statistically significant beneficial effect, non-statistically significant beneficial effect, no difference between control and intervention, non-significant detrimental effect or statistically significant detrimental effect (see Additional file 3)
Publication status	Published (Cochrane Library) [19]	Ongoing (protocol published in Cochrane Library) [21]	Ongoing (protocol published in Cochrane Library) [20]	Ongoing (protocol published on PROSPERO ref. CRD42015022078) [22]	Published (EPPI-Centre website) [23]

**Table 2:** Characteristics of included reviews which are reported in tables in overviews

Reported characteristics from included reviews	Pollock [19]	McClurg [21]	Estcourt [20]	Hunt [22]	Brunton [23]
Review reference	√	√	√	√	√
Date of search	√	√	√	√	
Country (of included studies)			√	√	√
Objective of review	√	√	√	√	
Types of studies included in review	√	√	√	√	√
Participants included in review	√	√	√	√	√
Intervention included in review (including name or brief description)	√	√	√		√
Reference standard(s) included in the review (including details: assessment tool used, version, assessor details, description)				√	
Index test(s) included in review (including brief cognitive assessment details: name, creator, version, description)				√	
Test thresholds included within the review				√	
Comparisons included in review	√	√	√	√	
Outcomes included in review	√	√	√		√
Target condition being addressed in the review				√	
Population prevalence of condition addressed in the review				√	
Setting focus of the review			√	√	√
Number of studies included in review	√	√	√	√	√
Number of participants included in review	√	√	√	√	
Other outcomes reported beyond diagnostic test accuracy in the review				√	
Statistical data from included reviews reported in overview					
Effect size	√	√	√		
Confidence intervals	√	√	√	√	
2 × 2 table components (TP, FP, TN, FN)				√	
Sensitivity, specificity				√	
Positive Predictive Value, Negative Predictive Value				√	
Tests compared, direct/indirect comparisons				√	
Heterogeneity (statistical measure)	√	√	√	√	
Direction of effect	√	√	√	√	√
Results of moderator analyses					√

### Key challenges within exemplar overviews and complementarity with published literature on overview methods

Discussion between overview authors led to agreement that there were a total of eight key methodological challenges encountered across all the exemplar overviews. These were the following:

**Table 3:** Summary of the perceived key methodological challenges associated with each of the exemplar overviews, a description of what the challenge was, and examples of how this challenge was dealt with within individual overviews

Key methodological challenges. Dealing with:	Description of challenge and why it was experienced	Complementarity with published literature on overview methods (Ballard [3])	Examples of how this challenge was dealt with within our exemplar overviews
a. Overlap between reviews (studies appearing in more than one review)	In overviews which include both Cochrane and non-Cochrane reviews, multiple published reviews were often identified which had similar aims and which included the same or similar trials.	Summary of findings from Ballard [3]:	Pollock [19] extracted details of the trials included within all relevant reviews, and explored which trials were included within which review. Reviews which were effectively superseded by a more comprehensive review of the same topic were excluded; the methods for making this judgement are described within the review [19]. 37 reviews which met the inclusion criteria but which were judged to be superseded by more up-to-date, comprehensive and methodologically rigorous reviews were excluded. These exclusions are transparently reported, and a table provided with characteristics of excluded reviews, including details of key characteristics as reported for included reviews (see Table 2).
		• “emerging debate related to (i) overlapping systematic reviews”	
		• Approaches to dealing with this challenge include:	
		o Calculation of degree of overlap using the “corrected cover area” (Pieper [2])	
		o Use “a priori criteria for choosing a single systematic review for inclusion when multiple potential candidates are available ”	
		o Use only Cochrane reviews (avoid overlap)	
	In overviews which included only Cochrane reviews, some trials were found to be included in more than one review. This was particularly the case for 3 or 4 arm trials, where different arms of the trial were included in different reviews.	• Optimal approach “currently remains unresolved.”	
			Hunt [22] identified a number of studies which were included in more than one systematic review, but did not exclude any systematic reviews on the basis of overlap. Where updated systematic reviews existed, the most recent review was selected over previous versions. Due to substantial heterogeneity across reviews, no meta-analysis or statistical synthesis was conducted and double counting of participants was not a risk. Hunt [22] explored the consistency of reporting of individual trial results within multiple reviews, and clearly highlighted any discrepancies which occurred.
		Judgement of complementarity	
		Agreement:	
		• A range of different approaches were used, and there was no consensus on an	
		• optimal approach	
		Silence (not raised by Ballard [3]):	
		• Two of our reviews (Hunt [22], McClurg [21]) included reviews which had overlapping trials, highlighting and reporting the overlap, but not taking further action	
			McClurg [21] only included Cochrane reviews, so the occurrences of overlapping reviews were reduced as compared to Pollock [19] or Hunt [22]. However there were still a number of trials identified within the reviews included in McClurg [21] which were included within two or more reviews. Details of the individual trials included in each review were extracted and occurrences of overlap systematically identified. Where a trial was found to occur in more than one review, details of that individual trial were obtained, and the existence of multiple treatment arms explored. In all occurrences of overlap the reviews were found to contain unique data from different active treatment arms, although control or comparison group data were often found to be included within more than one review. Details of these overlaps were documented and any times were there was multiple use of the same control group data were highlighted.
	If studies appear in more than one review then there are risks of double counting (where results of individual studies are included more than once within meta-analysis). This could be meta-analysis completed by overview authors, or completed by review authors and synthesised within the overview.		
		• McClurg 2016 [21] which only included Cochrane reviews still identified a problem with overlap – this conflicts with suggestions that inclusion of Cochrane reviews only is a method to avoid problems of overlap.	
		• Brunton 2016 [23] did not assess the overlap, stating that this was not important to the stated purpose of the overview.	
			Brunton 2016 [23] did not assess overlap between reviews; however this overview was designed as a ‘map of reviews’ rather than a full systematic review of reviews. The main purpose was to identify the characteristics that had been shown to be associated with larger effect sizes in order to compare those characteristics with those identified by stakeholder research and policy documents.
b. Reviews are out of date	Included reviews were judged to be out of date.	Summary of findings from Ballard [3]:	Estcourt [20] updated a number of reviews that were included. A judgement as to the relevance of each included reviews was applied, and updating was focused on only the reviews known to have included or ongoing trials. Potentially relevant RCTs that may require a review to be updated were identified using a broad search of the Transfusion Evidence Library (http://www.transfusionevidencelibrary.com/; a database, updated monthly, of systematic reviews (since 1980) and randomised controlled trials (since 1950) relevant to transfusion medicine, including grey literature. This database is officially endorsed by Cochrane). Estcourt [20] also searched the WHO Clinical trials database and Clinicaltrials.gov for relevant ongoing trials using a broad search strategy, with search terms: ‘sickle cell disease’ or ‘sickle cell an*emia’ and ‘transfusions’ or ‘blood transfusions’ or ‘red cell transfusions’. The decision was made that the overview authors would update most of the relevant reviews, this included reviews of red cell transfusions to prevent: development or progression of chronic lung disease [44]; sickle cell disease-related complications when having an operation [45]; prevention of a primary or secondary stroke [46]. Estcourt [20] also performed several new reviews on topics that did not have a Cochrane review, these included: prevention of chronic kidney disease [47]; and prevention of silent cerebral infarcts [48] [44–46] as well as performing the new reviews [47, 48]. The authors of the Cochrane review [49] agreed to update this. Another review in acute chest syndrome was already being updated [50], and the authors of another review agree to include red cell transfusion as an intervention when it is next updated but it was known that there are no relevant RCTs within that review [51].
		• “emerging debate related to (iv) updating included systematic reviews”	
		• Some guidelines ignore this issue, but approaches to deal with the challenge include:	
		o Updating included systematic reviews	
		o Searching for secondary and primary literature simultaneously	
		• Both approaches add complexity and time to the overview process.	
		• There is currently “no way to systematically investigate whether an update in the context of overviews is necessary .”	
		Judgement of complementarity	
		Agreement	
		• Estcourt [20] updated relevant reviews.	
		Other overview authors ignored this issue, only raising it during discussion.	
c. Definition of “systematic review”	Published review papers were identified which were described as “literature reviews” but did not meet expected methodological standards to be classed as a “systematic review”	Summary of findings from Ballard [3]:	Overview authors applied clear definitions of what a systematic review was during the stage of selecting relevant reviews of inclusion. Brunton [23] required that to be included a review had to describe (at a minimum) the search strategy, inclusion criteria and quality assessment methods.
		• Ballard [3] does not specifically identify the issue of the definition of a systematic review.	
		Judgement of complementarity	
		Silence (not raised by Ballard [3])	
		This challenge is raised by the overview authors but not identified in literature synthesised by Ballard [3].	
d. Assessment of methodological quality of reviews	Assessment of methodological quality using the AMSTAR was found to be challenging due to the multi-faceted nature of the questions within the AMSTAR tool. Assessment of methodological quality using the ROBIS was found to be challenging, with difficulties in reaching agreement between overview authors. There was no suitable tool available for assessing the methodological quality of reviews of diagnostic test accuracy.	Summary of findings from Ballard [3]:	The AMSTAR [42] and ROBIS [14] were the only two quality assessment tools used by our exemplar overviews:
		• “emerging debate related to (iii) evaluating the quality and reporting of included research”	
		• (systematic reviews)	Pollock [19] developed and implemented a modified version of the AMSTAR.
		• There is no consensus on what instrument should be used to assess methodological quality of systematic reviews	
			Estcourt [20] used AMSTAR but added explanatory text to explain reason authors made decision.
		• Many overviews don’t assess methodological quality of systematic reviews (Hartling [1]) and there are a “diversity of instruments” used	
			McClurg [21] used ROBIS, providing explanatory text to explain reason authors made decision. Independent authors within the McClurg [21] overview experienced difficulties interpreting the signalling questions used to prompt judgements relating to the four domains of phase 2 of the ROBIS tool, which led to high levels of disagreement and the need for substantial discussion, and the involvement of an arbitrator. During discussion amongst three overview authors there were several areas of continued disagreement, and this contributed to a post-hoc decision not to report a final overall judgement of risk of bias for each review [21].
		Judgement of complementarity	
		Agreement	
		• A range of tools were used.	
		Silence (not raised by Ballard [3])	
		• Challenges in gaining agreement in ROBIS judgements between review authors were identified by overview authors.	
			Hunt [22] suggests modifications to the AMSTAR and ROBIS tools in order to fit within a diagnostic test accuracy review framework.
e. Quality of reporting within reviews	Methodological assessments, using either the AMSTAR or ROBIS, were limited due to the quality of reporting of the reviews. It was therefore challenging to determine whether the scores provided by AMSTAR or ROBIS reflected the quality of the methods or quality of the reporting.	Summary of findings from Ballard [3]:	Pollock [19] changed the last question of the AMSTAR, so that it was a judgement on quality rather than on presence of information.
		• “emerging debate related to (iii) evaluating the quality and reporting of included research” (systematic reviews)	
		• Important to differentiate between methodological quality and reporting quality.	
			McClurg [21] and Estcourt [20] added explanatory text to justify decisions made by authors.
		Judgement of complementarity	
		Agreement	Hunt [22] reported that the quality of reporting in DTA studies was generally found to be poor, and this was observed in this overview where quality of reporting was found to be mixed. Cochrane DTA reviews were of a higher quality than non-Cochrane DTA reviews.
		• Overview authors attempted to differentiate between methodological quality and reporting quality.	
		Silence (not raised by exemplar overviews)	
		• To address reporting issues Ballard [3] “recommended that PRISMA be used in conjunction with a comprehensive, validated critical appraisal tool.”	
f. Applying GRADE	The GRADE approach has been developed specifically for judgements of quality of evidence during guideline development, and also adopted for judgement of quality of evidence within Cochrane reviews [33]. There is an absence of guidance on how to apply GRADE within an overview. Authors using GRADE faced challenges relating to the number of comparisons, and subtle differences between comparisons, which created issues in terms of workload and in relation to achievement consistency.	Summary of findings from Ballard [3]:	Pollock [19] identified challenges in consistent application of the GRADE approach to large volumes of evidence synthesised within overviews [37], and proposedla more algorithmic approach to judging quality of evidence within reviews [10]. There remains debate about the validity of this proposed approach [11, 12], but Pollock [19] argues that approach does arguably facilitate transparency and consistency when faced with judging the quality of evidence of many similar (but not identical) comparisons included within reviews.
		• “emerging debate related to (iii) evaluating the quality and reporting of included research” (“quality of the body of evidence across included systematic reviews”)	
		• GRADE has been described as an approach for assessing the quality of the body of evidence accrsoss systematic reviews, but there is currently a lack of guidance to ensure appropriate use and interpretation of GRADE when applied in this way	
			McClurg [21] repeated this method and developed algorithm using the method recommended by Pollock [19], but involving a wider group of people in the decision making, including statisticians and clinicians.
		Judgement of complementarity	
		Agreement	Estcourt [20] used GRADE levels of evidence from within included reviews. All new and updated reviews that included relevant comparisons had performed GRADE assessments. The participants within the reviews differed (pregnancy, preoperative, high risk of stroke etc.). Therefore, individual GRADE assessments of an outcome had to be reported for a particular type of participant.
		• Overview authors used GRADE to explore quality of evidence, identifying absence of guidance of how to apply this within the context of an overview.	
g. Potential for publication bias	The potential for publication bias comes from two sources – publication bias relating to the identification and inclusion of relevant reviews, and publication bias relating to the trials which are identified and included within the reviews.	Summary of findings from Ballard [3]:	Other than being transparent about the potential risks of publication bias, no additional action was undertaken within our overviews. Hunt [22] highlighted particular challenges relating to the exploration of publication bias relating to reviews of diagnostic test accuracy and due to lack of consensus in handling publication bias within DTA reviews this was not assessed (as pre-stated in the protocol).
		• While the issue of publication/reporting bias is not explicitly raised as a methodological challenge within guidance on overview methods, Ballard [3] concludes that “overviews are always susceptible to….and reporting biases”	
		• Where systematic reviews are at high risk of reporting biases then a systematic review (rather than overview) may produce the most precise result	
		Judgement of complementarity	
		Agreement	
		The issue of publication bias is raised by both Ballard [3] and our exemplar overviews, although no specific actions to address or alleviate these biases are identified.	
h. Summarising key findings in brief accessible format, suitable for informing decision making	A brief, often single page, summary has insufficient space to address the factors that decision makers require to inform clinical or policy decisions. Decision makers require details of what works, with whom and in what way.	Summary of findings from Ballard [3]:	Pollock [19] and McClurg [21] highlight that the aim of the overview is to signpost clinical decision makers to the reviews which are best placed to inform decisions, and clearly state that the overview does not provide sufficient evidence to make individual treatment decisions. Pollock [19] produced a single page summary table, incorporating a traffic light system to indicate evidence of effectiveness, summarising interventions for which there is evidence of effectiveness in relation to specific outcomes, and stating the quality of that evidence.
		• “emerging debate related to (v) synthesising and reporting the result of included systematic reviews”	
		• Functions of an overview can be to explore heterogeneity or summarise evidence.	
			Brunton [23] integrated the results of an overview of reviews with a mixed method synthesis relating to stakeholder views and key policy documents. A single page structured summary was produced confirming interventions which are supported by evidence, and stating that specific characteristics relating to the intervention implementation may be important. .
		Judgement of complementarity	
		Agreement	
		• Our overviews aimed to summarise evidence.	Hunt [22] is conducting additional analysis of outcomes, subsequent to completion of the overview of reviews of diagnostic test accuracy, in order to more fully inform decision making.
			Within protocols, McClurg 2016 [21] proposed use of a matrix to summarise research findings and Estcourt [20] proposes the use of the template provided within the Cochrane Handbook for summarising findings within overview (Additional file 1).

- Overlap between reviews (studies appearing in more than one review)
- Reviews are out of date
- Definition of “systematic review”
- Assessment of methodological quality of reviews
- Quality of reporting within reviews
- Applying GRADE
- Potential for publication bias
- Summarising key findings in brief accessible format, suitable for informing decision making

The judgement of complementarity between these identified methodological challenges and the areas of debate identified within current literature are summarised in Table 3 and below.

### Complementarity with published literature

- *Agreement*: seven of our identified key methodological challenges were in agreement with the issues raised by Ballard [3]; although, some additional specific points (categorised as areas of dissonance) were raised by our exemplar overviews in relation to three of the issues (see Table 3 for details)
- *Silence*:
  - o Methodological challenge highlighted by our overviews, but not raised by Ballard [3]): there was one area of silence, with our exemplar overviews identifying challenges relating to gaining agreement with the ROBIS tool. (Note: the ROBIS tool was published after the search period of Ballard [3])
  - o Methodological issue identified by Ballard [3] but not from our exemplar overviews: there were two areas of silence. These included challenges relating to the “scope of systematic reviews”, where there is a “mismatch” between the scope of the systematic review and the remit or focus of the overview, and challenges associated with the assessment of risk of bias of primary trials, where appropriate quality assessment was not used within the systematic review
- *Dissonance*: there were no areas of dissonance between the methodological issues raised by our overviews and Ballard [3]. However, there was disagreement noted in relation to conclusions drawn by Ballard [3] relating to the function of overviews and that overviews cannot identify evidence gaps: three of our exemplar overviews [19, 21, 23] clearly concluded that the overview had successfully identified gaps in the evidence.

### Challenges common to systematic reviews

Also common to all exemplar overviews were a number of challenges which were considered to be routine amongst most systematic reviews. These included challenges such as the management of large volumes of review information and data extraction, the available time and resources, and reaching consensual decisions amongst overview authors. Strategies implemented to address these challenges included methods of automating data extraction (for example downloading data files for Cochrane reviews) and audio-recording discussions between overview authors.

### Implications for development of protocols for future overviews

The identified common methodological features, implications for future overviews and implications for development of protocols for future overviews are summarised in Table 4.

**Table 4:** Summary of common features and differences between the exemplar overviews, and implications for development of protocols for future overviews

Common features	Differences	Implications for development of protocols for future overviews
Clearly defined aim or question.	The aim or question can vary considerably depending on the focus of the overview.	1. Be clear about the purpose of the overview and ensure clear definition of aim or question.
		2. Involve key stakeholders at the development stage to ensure the planned overview is relevant and useful. Resources being compiled by Cochrane Training will inform methods of involving people [35, 36].
Includes relevant reviews.	The reviews may be reviews of randomised or quasi-randomised trials, or may be reviews of other study designs, including non-randomised, qualitative or mixed methods studies.	3. Ensure clear definition and justification of scope of overview.
		4. Include a clear definition of what criteria constitute a “systematic review”. These criteria may include; a pre-defined protocol detailing the scope and methods to be employed in conducting the review; a specific and detailed search strategy giving at least one example of the exact terms used within the search; a clear selection process in line with pre-specified criteria for inclusion in the systematic review; assessment of included studies for various types of bias including publication bias; synthesis of research findings in order to address the original research question; discussion of results in relation to existing evidence, limitations of the research and included studies and contribution to the field of study [52, 53].
	Some Cochrane overviews only include Cochrane systematic reviews, whilst other Cochrane overviews include any systematic reviews.	
	Some overviews include primary research studies in addition to review. Additional searches for new primary studies have been considered when identified systematic review evidence has been judged to be out of date, or there has been known gaps within systematic review evidence.	
		5. Plan, and clearly state within the overview protocol, how multiple overlapping reviews (i.e. systematic reviews which address the same, or similar, research questions, including the same, or similar, primary research studies [54] will be dealt with. Consensus is yet to be reached on the best way to deal with overlapping review evidence, but there is a clear need for guidance and improved transparency in the way in which authors deal with this problem [55].
		6. Clearly state plans for action to be taken if identified systematic review evidence is out of date or gaps in the evidence (i.e. absence of reviews) are identified) [56]. If searching for primary studies is to be considered, full details of the scope and methods for this should be provided. Consider contacting authors of original reviews to see if they would update this. Note: Pieper [25] proposes and discusses two different approaches for searching for primary studies to ensure an overview is up-to-date; one approach involves searching for reviews and primary studies in parallel, whilst the other approach involves identifying the most up-to-date review and updating the searches from the date of the last search [56].
A clearly defined selection criteria for included reviews.	The parameters or domains which are defined reflect the aims/focus of the overview.	7. Ensure definition of selection criteria which are relevant to the aims/focus of the overview.
A structured search strategy to identify relevant reviews	The search strategy may be limited to databases of reviews (e.g. CDSR, DARE^a^), or may involve searching a wide variety of electronic databases.	8. Consider and justify whether overview is limited to Cochrane reviews only or whether non-Cochrane reviews are to be included, with reflection on the available time and resources, and the potential added value of including non-Cochrane reviews and searching additional databases. If non-Cochrane reviews are to be included, state databases to be searched. With the discontinuation of the DARE database, authors should consider which key databases to search, the potential added value of searching additional databases, and the use of a validated search filter for systematic reviews. Authors should remain aware of any new database options, as there is ongoing work to develop comprehensive databases of systematic reviews (e.g. EPISTEMONIKOS [57] and PDQ-Evidence [58].
	The search strategy may have date restrictions. The use of date restrictions has been proposed as an approach to searching for literature within limited timescales, justification provided often relates to improvement in the quality and consistency of systematic review evidence over time (including improvements from 1999 following release of the QUality Of Reporting Of Meta-analyses (QUOROM) [31] statement was released.	
Two independent overview authors apply inclusion criteria and select reviews.	–	9. Plan for two independent overview authors to apply inclusion criteria and select reviews.
Systematic extraction and reporting of key characteristics of included reviews.	–	10. Clearly state data to be extracted, including the characteristics of included reviews to be extracted.
Systematic extraction and reporting of results of meta-analyses	Some overviews extract and report key statistical data (e.g. effect size, confidence intervals), whilst others only summarise the direct of effect.	11. Clearly state the statistical data which is to be extracted, and how this will be summarised. Detail any re-analysis if this is planned.
	Some overviews carry out some degree of re-analysis of the results presented in the reviews.	
Assessment of methodological quality of included reviews.	The tool which is used to assess methodological quality or risk of bias varies, and may be the AMSTAR or ROBIS tool. The method of applying and reporting the results from these tools can vary.	12. Select and justify a tool to assess methodological quality of included reviews, according to up-to-date evidence relating to available tools, and with consideration of the need to assess quality of methods and/or risk of bias. Note: The issues raised in relation to the AMSTAR have been debated by other authors [59–61], and the use of a priori decision tools has been proposed as a method to support the valid, reliable use of the AMSTAR tool within overviews [13].
		13. Plan to have two independent reviewers assessing methodological quality, and state how any disagreements will be addressed.
		14. Plan to report the results of individual questions or domains (not just a summary score).
Assessment of quality of evidence within reviews.	If quality of evidence within reviews is assessed the GRADE approach (for reviews of effectiveness) and the QUADAS-2 [39] (for reviews of diagnostic test accuracy) have been used. The method of applying the GRADE approach varies between overviews, and an algorithmic approach has been explored.	15. Consider best evidence and guidance relating to the application of tools to assess quality of evidence within reviews. Be aware of new developments and guidance in this field, and build on methods in previous overviews.
Data synthesis and summary of key findings.	While the need for clear, accessible summaries of key findings is common across all overviews, the methods of summarising key findings vary.	16. Clearly state how key findings will be summarised, with consideration of summarising the findings in relation to the quality of evidence (e.g. GRADE), and the populations, interventions and outcomes which this evidence relates to (for evidence relating to effectiveness), and the audience to whom the overview is of relevance. A number of different templates for summarising the findings of an overview have been proposed, including simple ‘traffic light’ graphics to illustrate evidence of effective, non-effective or detrimental interventions [4] and structured summary of results tables [8, 62, 63].

^a^DARE is no longer being maintained, meaning that this database will not be up-to-date.

## Discussion

Using five recently completed or ongoing overviews of reviews, we have explored the methodological features of overviews and the key methodological challenges which are reported by the overview authors and have systematically compared these findings with synthesised evidence relating to current guidance for overview methods (Ballard [3]). Our five overviews provide examples of a range of different types of overviews, including Cochrane overviews, mixed method overviews and overviews of reviews of diagnostic test accuracy. There are some methodological differences between our exemplar overviews which cannot be attributed to the type or aim of the overviews, and these arguably occur due to lack of information and guidance on the optimal methods for overviews and reinforce recent calls for methodological research and improved guidance for overviews [2, 3, 5, 24, 25].

Eight key methodological challenges were encountered during our five exemplar overviews; these challenges were clearly aligned with the areas of debate identified within current literature [3], with no areas of dissonance. Areas of silence where a methodological issue was identified by our exemplar overviews and not by the synthesis by Ballard, and vice versa, can arguably be explained by differing levels of reporting within these syntheses. Where there were methods debated within our exemplar overviews which were not discussed in the synthesis by Ballard [3], these were always related to a larger topic or issue for which there was no consensus on an optimal approach, for example dealing with overlap between reviews.

Our exemplar overviews supported the conclusion that overviews can successfully identify gaps in the evidence; this conflicts with Ballard [3] who concludes that overviews cannot fulfil the function of identifying evidence gaps. However, Ballard [3] qualifies this, stating that “overviews that fail to find a systematic review for every relevant comparison will not, by default, detect evidence gaps”. Our experiences suggest that there are situations where evidence gaps will be identified, specifically when there is documented knowledge of current clinical practice or existing interventions. For example, during protocol development McClurg [21] consulted with a stakeholder group comprising expert clinicians and patients, creating a comprehensive list and taxonomy of interventions delivered within clinical practice relevant to the scope of the overview. While Ballard implies that overviews cannot fulfil the function of identifying evidence gaps, we argue that this function can be fulfilled, where there is knowledge of existing interventions or current clinical practice. This supports the viewpoint that the involvement of key stakeholders within the overview process should be an essential component of overview methodology, serving to increase relevance, quality and rigor and reduce research waste, as has been proposed for systematic reviews [26–30].

Our experiences as authors completing overviews, despite an absence of guidance for many methodological features, and the evidence of the increasing number of new overviews published each year [1, 2] highlight that there is a need for recommendations and support for authors wishing to complete overviews during this time of methodological uncertainty. Clearly, the ultimate goal must be to address the uncertainties and establish evidence-based guidance for overview methods, and there is ongoing work which aims to inform and help prioritise future research in this field [15]. However, while there remains a lack of guidance and recommendations for optimal methods, there is currently decision-making required by the authors of future overviews. Until sufficient guidance is available, we recommend that overview authors make and transparently report decisions relating to the inevitable methodological choices. Based on our experiences as overview authors, we have proposed a list of implications to be considered during the development of protocols for future overviews (Table 4). Whilst we anticipate that these implications will be superseded by more robust and evidence-based recommendations as methodological research in this field is completed, we believe that these offer practical advice to those embarking on overviews whilst there remains methodological uncertainty. As this is an active area of research and the methods for overviews continue to develop, overview authors should ensure that they remain up-to-date with any new guidance or information on best practice and should take opportunities to build on the methods of completed overviews. Furthermore, as the methodological features of overviews are broadly derived from, and build upon, methods for systematic reviews of primary research [1, 4, 5], overview authors should utilise guidance and recommendations relating to the conduct of systematic reviews [1]. These include the QUality Of Reporting Of Meta-analyses (QUOROM) statement [31], Methodological Expectations of Cochrane Intervention Reviews guidance (MECIR) [32] and Preferred Reporting Items for Systematic Reviews and Meta-Analyses (PRISMA) guidelines, as well as the growing body of guidance specific to overviews [1, 4, 33, 34]. As well as ensuring the overview is carried out to the highest methodological standards, it is essential to ensure that the overview is relevant and useful, and meaningful involvement of key stakeholders should be central to all overviews (Hunt H, Pollock A, Campbell P, Estcourt L, Brunton G: An introduction to overviews of reviews: planning a relevant research question and objective for an overview Systematic Reviews, submitted); resources currently being developed to support systematic review authors in achieving meaningful stakeholder involvement [35, 36] ought to be relevant to authors of overviews.

### Strengths and limitations

The aim of this paper was to provide illustrated examples of the methods and challenges associated with a range of overviews. The objective was not to provide specific recommendations about how to do an overview, or to propose optimal methods for overviews. The five exemplar overviews were initially selected using contacts for overview authors whom attended a meeting on overviews at the 2015 Cochrane Colloquium in Vienna. As the exemplar overviews were initially selected for presentation at the 2016 UK Cochrane meeting, all the selected overviews were led by UK-based authors. Two of the exemplar overviews include some of the same authors [19, 21]. Thus, the overviews provided as examples in this paper are selected from a limited population of authors and cannot therefore be assumed to be representative or comprehensive of the range of different overviews which have been published internationally. None of the five exemplar overviews addresses a research question for which there was high quality trial and review evidence and none of these exemplar overviews completed network meta-analyses; these are clear gaps within these example overviews. However, whilst not purporting to be comprehensive, the examples reflect the shared challenges experienced by the authors of these five selected overviews, and those which were perceived to be the greatest or most difficult to deal with. The comparison of the methodological challenges independently identified from these exemplar overviews with the issues emerging from a comprehensive synthesis of current evidence adds significant strength to this paper, confirming that the experiences within these exemplar overviews are aligned with current evidence.

## Conclusions

Overviews are a relatively new methodological innovation, and there are currently substantial variations in the methodological approaches used within different overviews. Furthermore, there are considerable methodological challenges for which optimal solutions are not necessarily yet known. This paper has explored the variations in methodological approaches used within five selected overviews, and the challenges reported by the overview authors. Lessons learnt from these overviews have highlighted a number of methodological decisions which may need to be considered during the development of an overview protocol and led to the development of a list of implications to support the development of protocols for future overviews (Table 4).

While there remains a lack of empirical evidence to support selection of specific methodological approaches [1–3, 5, 6], authors planning protocols for Cochrane overviews are encouraged to consider and transparently report their decisions in response to a number of questions, including the following:

- Is the overview to be limited to Cochrane reviews or will other systematic reviews also be included?
- Is the search strategy to be limited to databases of reviews or will wider electronic databases be searched?
- What action will be taken if there are overlapping reviews (reviews containing the same trials)?
- What action will be taken if included reviews are out of date? (How will ‘out of date’ be defined?)
- What tool will be used to assess the quality of the included reviews? (AMSTAR/ROBIS)
- How will the quality of evidence within reviews be assessed? Will this be done using GRADE (and if so, how will GRADE be applied to the available evidence)?
- Is any new statistical analysis to be carried out, using the data extracted from the reviews?
- How will the evidence be brought together into an accessible summary which is useful to the potential audience/readership of the overview? What information should be included within this (in order to address the overview objective)?

As this is a new and developing methodological field, it is important that overview authors keep up-to-date with new developments and methods research, in order that their decisions relating to the methodological approach for a new overview are informed by current evidence.

## Additional files

Additional file 1: Summary of findings as presented by Pollock [8]. (DOCX 777 kb)

Additional file 2: Summary of findings as presented by Brunton [12]. (DOCX 20 kb)

Additional file 3: Summary of results tables proposed by McClurg [10] and Escourt [9]. (DOCX 22 kb)

## References

[1] Hartling L, Chisholm A, Thomson D, Dryden DM (2012). A descriptive analysis of overviews of reviews published between 2000 and 2011. PLoS ONE.

[2] Pieper D, Buechter R, Jerinic P, Eikermann M (2012). Overviews of reviews often have limited rigor: a systematic review. J Clin Epidemiol.

[3] Ballard M, Montgomery P (2017). Risk of bias in overviews of reviews: a scoping review of methodological guidance and four-item checklist. Research Synthesis Methods.

[4] Aromataris E, Fernandez R, Godfrey CM, Holly C, Khalil H, Tungpunkom P (2015). Summarizing systematic reviews: methodological development, conduct and reporting of an umbrella review approach. Int J Evid Based Healthc.

[5] Pollock M, Fernandes R, Becker L, Featherstone R, Hartling L (2016). What guidance is available for researchers conducting overviews of reviews of healthcare interventions? A scoping review and qualitative metasummary. Systematic Reviews.

[6] Thomson D, Foisy M, Oleszczuk M, Wingert A, Chisholm A, Hartling L (2013). Overview of reviews in child health: evidence synthesis and the knowledge base for a specific population. Evidence-Based Child Health: A Cochrane Review Journal.

[7] Caird J, Sutcliffe K, Kwan I, Dickson K, Thomas J. Mediating policy-relevant evidence at speed: Are systematic reviews of systematic reviews a useful approach? Evidence and Policy. 2015;11:81-97.

[8] Smith V, Devane D, Begley CM, Clarke M (2011). Methodology in conducting a systematic review of systematic reviews of healthcare interventions. BMC Med Res Methodol.

[9] Pollock A, Hunt H, Campbell P, Estcourt L, Brunton G (2016). Cochrane overviews of reviews: exploring the methods and challenges.

[10] Pollock A, Farmer SE, Brady MC, Langhorne P, Mead GE, Mehrholz J, Van Wijck F, Wiffen PJ (2016). An algorithm was developed to assign GRADE levels of evidence to comparisons within systematic reviews. J Clin Epidemiol.

[11] Murad MH, Mustafa R, Morgan R, Sultan S, Falck-Ytter Y, Dahm P (2016). Rating the quality of evidence is by necessity a matter of judgment. J Clin Epidemiol.

[12] Gionfriddo MR (2016). Subjectivity is a strength: a comment on “an algorithm was developed to assign GRADE levels of evidence to comparisons within systematic reviews”. J Clin Epidemiol.

[13] Pollock M, Fernandes RM, Hartling L (2017). Evaluation of AMSTAR to assess the methodological quality of systematic reviews in overviews of reviews of healthcare interventions. BMC Med Res Methodol.

[14] Whiting P, Savovic J, Higgins JP, Caldwell DM, Reeves BC, Shea B, Davies P, Kleijnen J, Churchill R, group R (2016). ROBIS: a new tool to assess risk of bias in systematic reviews was developed. J Clin Epidemiol.

[15] Lunny C, Brennan SE, McDonald S, McKenzie JE (2016). Evidence map of studies evaluating methods for conducting, interpreting and reporting overviews of systematic reviews of interventions: rationale and design. Systematic Reviews.

[16] Pollock M, Hartling L: Preferred reporting items for overviews of reviews (PRIOR). EQUATOR Network; 2016. http://www.equator-network.org/library/reporting-guidelines-under-development/#72. 10.1186/s13643-019-1252-9 PMC6929355 31870434

[17] Erzberger C, Prein G (1997). Triangulation: validity and empirically-based hypothesis construction. Qual Quant.

[18] O’Cathain A, Murphy E, Nicholl J. Three techniques for integrating data in mixed methods studies. BMJ. 2010;341. 10.1136/bmj.c4587 20851841

[19] Pollock A, Farmer SE, Brady MC, Langhorne P, Mead GE, Mehrholz J, Van Wijck F (2014). Interventions for improving upper limb function after stroke. Cochrane Database Syst Rev.

[20] Estcourt LJ, Fortin PM, Hopewell S, Trivella M: Red blood cell transfusion to treat or prevent complications in sickle cell disease: an overview of Cochrane reviews. *Cochrane Database Syst Rev* 2016, 2016. 10.1002/14651858.CD012082 PMC4826604 27069421

[21] McClurg D, Pollock A, Campbell P, Hazelton C, Elders A, Hagen S, Hill DC, McClurg D: Conservative interventions for urinary incontinence in women: an Overview of Cochrane systematic reviews. *Cochrane Database of Systematic Reviews* 2016. 10.1002/14651858.CD012337.pub2 PMC9437962 36053030

[22] Hunt H, Kuzma E, C H: A review of existing systematic reviews summarising the accuracy of brief cognitive assessments for identifying dementia, particularly for use in primary care. Protocol..In *PROSPERO* PROSPERO online; 2016.

[23] Brunton G, Dickson K, Khatwa M, Caird J, Oliver S, Hinds K, Thomas J (2016). Developing evidence-informed, employer-led workplace health.

[24] Li L, Tian J, Tian H, Sun R, Liu Y, Yang K (2012). Quality and transparency of overviews of systematic reviews. Journal of Evidence-Based Medicine.

[25] Pieper D, Antoine S-L, Morfeld J-C, Mathes T, Eikermann M (2014). Methodological approaches in conducting overviews: current state in HTA agencies. Research Synthesis Methods.

[26] Boote J, Wong R, Booth A (2015). ‘Talking the talk or walking the walk?’ A bibliometric review of the literature on public involvement in health research published between 1995 and 2009. Health Expect.

[27] INVOLVE (2012). Public involvement in systematic reviews: Supplement to the briefing notes for researchers.

[28] Kreis J, Puhan MA, Schunemann HJ, Dickersin K (2013). Consumer involvement in systematic reviews of comparative effectiveness research. Health Expect.

[29] Morley R, Norman G, Golder S, Griffith P (2016). A systematic scoping review of the evidence for consumer involvement in organisations undertaking systematic reviews: focus on Cochrane. Research Involvement and Engagement.

[30] Serrano-Aguilar P, Trujillo-Martin MM, Ramos-Goni JM, Mahtani-Chugani V, Perestelo-Perez L, Posada-dela Paz M (2009). Patient involvement in health research: a contribution to a systematic review on the effectiveness of treatments for degenerative ataxias. Soc Sci Med.

[31] Moher D, Cook DJ, Eastwood S, Olkin I, Rennie D, Stroup DF (1999). Improving the quality of reports of meta-analyses of randomised controlled trials: the QUOROM statement. Quality of Reporting of Meta-analyses. Lancet.

[32] MECIR (2017). The Methodological Expectations of Cochrane Intervention Reviews (MECIR).Standards for Cochrane new reviews of interventions and their updates.

[33] Becker L, Oxman A: Chapter 22: Overviews of reviews. In *Cochrane Handbook for Systematic Reviews of Interventions Version 510* (JPT H, S G eds.): The Cochrane Collaboration; 2011. Available from www.handbook.cochrane.org.

[34] Conn VS, Sells TGC (2014). WJNR Welcomes Umbrella Reviews. West J Nurs Res.

[35] Pollock A, Campbell P, Struthers C, Synnot A, Nunn J, Hill S, Goodare H, Watts C, Morley R (2017). Stakeholder involvement in systematic reviews: a protocol for a systematic review of methods, outcomes and effects. Research Involvement and Engagement.

[36] ACTIVE: Authors and consumers together impacting on evidence [http://training.cochrane.org/ACTIVE]

[37] Pollock A, Brady MC, Farmer SE, Langhorne P, Mead GE, Mehrholz J, Wiffen PJ, Van Wijck F (2016). The purpose of rating quality of evidence differs in an overview, as compared to guidelines or recommendations. J Clin Epidemiol.

[38] Bossuyt PM, Reitsma JB, Bruns DE, Gatsonis CA, Glasziou PP, Irwig LM, Moher D, Rennie D, De Vet HC, Lijmer JG, Standards for Reporting of Diagnostic A (2003). The STARD statement for reporting studies of diagnostic accuracy: explanation and elaboration. Ann Intern Med.

[39] Whiting PF, Rutjes AW, Westwood ME, Mallett S, Deeks JJ, Reitsma JB, Leeflang MM, Sterne JA, Bossuyt PM, Group Q (2011). QUADAS-2: a revised tool for the quality assessment of diagnostic accuracy studies. Ann Intern Med.

[40] PSTF (2008). U.S. Preventive Services Task Force. Procedure Manual. AHRQ publication no 08-05118-EF.

[41] NICE (2006). National Institute for Health and Clinical Excellence. The Guidelines Manual.

[42] Shea BJ, Grimshaw JM, Wells GA, Boers M, Andersson N, Hamel C, Porter AC, Tugwell P, Moher D, Bouter LM (2007). Development of AMSTAR: a measurement tool to assess the methodological quality of systematic reviews. BMC Med Res Methodol.

[43] NOS (2013). Newcastle-Ottawa Scale (NOS) for assessing the quality of nonrandomised studies in meta-analyses.

[44] Estcourt LJ, Fortin PM, Hopewell S, Trivella M, Hambleton IR, Cho G (2016). Regular long-term red blood cell transfusions for managing chronic chest complications in sickle cell disease. Cochrane Database Syst Rev.

[45] Estcourt LJ, Fortin PM, Trivella M, Hopewell S (2016). Preoperative blood transfusions for sickle cell disease. Cochrane Database Syst Rev.

[46] Estcourt LJ, Fortin PM, Hopewell S, Trivella M, Wang WC (2017). Blood transfusion for preventing primary and secondary stroke in people with sickle cell disease. Cochrane Database Syst Rev.

[47] Roy NB, Fortin PM, Bull KR, Doree C, Trivella M, Hopewell S, Estcourt LJ: Interventions for chronic kidney disease in people with sickle cell disease. In *Cochrane Database of Systematic Reviews*: John Wiley & Sons, Ltd; 2016. 10.1002/14651858.CD012380 PMC5360229 28344511

[48] Estcourt LJ, Fortin PM, Hopewell S, Trivella M, Doree C, Abboud MR: Interventions for preventing silent cerebral infarcts in people with sickle cell disease. In *Cochrane Database of Systematic Reviews*: John Wiley & Sons, Ltd; 2016. 10.1002/14651858.CD012389 PMC5360228 28344510

[49] Okusanya BO, Oladapo OT (2013). Prophylactic versus selective blood transfusion for sickle cell disease in pregnancy. Cochrane Database Syst Rev.

[50] Dastgiri S, Dolatkhah R: Blood transfusions for treating acute chest syndrome in people with sickle cell disease. In *Cochrane Database of Systematic Reviews*: John Wiley & Sons, Ltd; 2016. 10.1002/14651858.CD007843.pub3 27574910

[51] Martí-Carvajal AJ, Knight-Madden JM, Martinez-Zapata MJ: Interventions for treating leg ulcers in people with sickle cell disease. In *Cochrane Database of Systematic Reviews*: John Wiley & Sons, Ltd; 2014. 10.1002/14651858.CD008394.pub3 PMC7175837 25485858

[52] Gough D, Oliver S, Thomas J: Learning from research: systematic reviews for informing policy decisions (a quick guide). http://www.alliance4usefulevidence.org/assets/Alliance-FUE-reviews-booklet-3.pdf: EPPI-Centre, Social Science Research Unit, Institute of Education, University of London; 2013.

[53] CCN: What is a systematic review? http://consumers.cochrane.org/what-systematic-review: Cochrane consumers network (CCN); 2017.

[54] Pieper D, Antoine SL, Mathes T, Neugebauer EA, Eikermann M (2014). Systematic review finds overlapping reviews were not mentioned in every other overview. J Clin Epidemiol.

[55] Ioannidis JP (2016). The mass production of redundant, misleading, and conflicted systematic reviews and meta-analyses. Milbank Q.

[56] Pieper D, Antoine SL, Neugebauer EA, Eikermann M (2014). Up-to-dateness of reviews is often neglected in overviews: a systematic review. J Clin Epidemiol.

[57] Epistemonikos: Epistemonikos: Database of the best evidence-based health care. In http://www.epistemonikos.org/; 2017.

[58] PDQ-Evidence: PDQ-Evidence for informed health policymaking. In http://www.pdq-evidence.org; 2017.

[59] Burda BU, Holmer HK, Norris SL (2016). Limitations of A Measurement Tool to Assess Systematic Reviews (AMSTAR) and suggestions for improvement. Systematic Reviews.

[60] Faggion CM (2015). Critical appraisal of AMSTAR: challenges, limitations, and potential solutions from the perspective of an assessor. BMC Med Res Methodol.

[61] Wegewitz U, Weikert B, Fishta A, Jacobs A, Pieper D (2016). Resuming the discussion of AMSTAR: what can (should) be made better?. BMC Med Res Methodol.

[62] EPOC (2016). Effective Practice and Organisation of Care (EPOC): reporting the effects of an intervention in EPOC reviews. Section 24. How to report the effects of an intervention. EPOC Resources for review authors.

[63] Glenton C, Santesso N, Rosenbaum S, Nilsen ES, Rader T, Ciapponi A, Dilkes H (2010). Presenting the results of Cochrane Systematic Reviews to a consumer audience: a qualitative study. Med Decis Making.

