# Supplementary material for: Selecting and implementing overview methods: implications from five exemplar overviews
Source: Syst Rev. 2017 Jul 18;6:145. doi: 10.1186/s13643-017-0534-3 (PMC5516331; doi:10.1186/s13643-017-0534-3)
Supplement: Supplementary file 1 — Summary of findings as presented by Pollock [8]. (DOCX 777 kb) [file 13643_2017_534_MOESM1_ESM.docx]

**Additional file 1. Summary of findings as presented by Pollock 2014** [8]

**Figure 2 from Pollock 2014: “Summary of findings”**

**
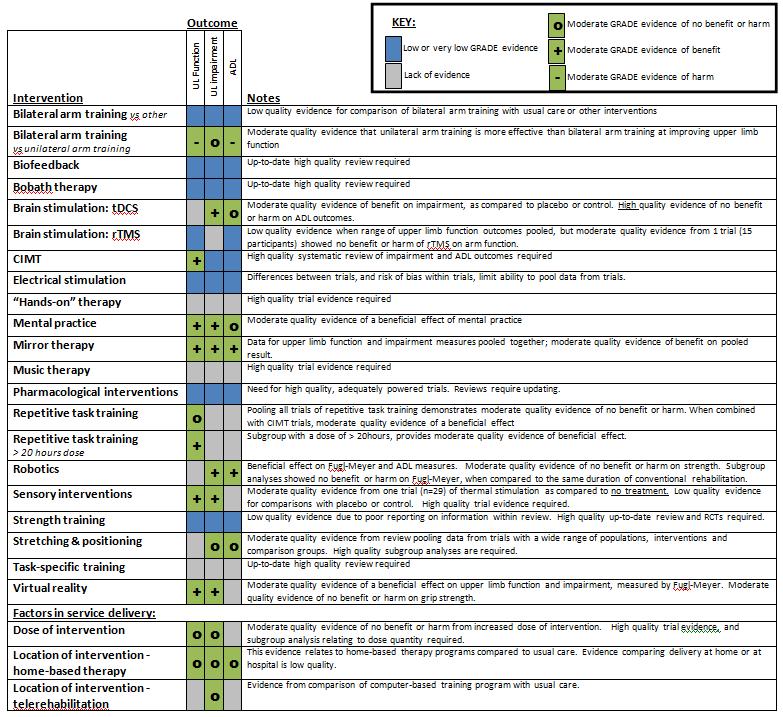
**

**Table 2 from Pollock 2014: “Summary of results and implications related to individual interventions.”**

| **Intervention** | **Included reviews** | **Moderate-quality evidence of effect on upper limb function** | **Moderate-quality evidence of effect on upper limb impairment** | **Moderate-quality evidence of effect on ADL outcomes** | **Low- or very low-quality evidence** | **Implications for clinical practice** | **Recommendations for research** |
| --- | --- | --- | --- | --- | --- | --- | --- |
| **Bilateral arm training** | **Coupar 2010** (vs usual care or control)  **van Delden 2012** (vs unilateral arm training) | **Unilateral arm training more effective** than bilateral arm training  (6 trials, n = 375) | **No difference**  between unilateral arm training and bilateral arm training (4 trials, n = 228) | **Unilateral arm training more effective** than bilateral arm training | Low-quality evidence for bilateral arm training compared with usual care or other interventions | Evidence does not support bilateral arm training as a replacement for unilateral arm training | A sound theoretical rationale is essential to justify further research into bilateral arm training |
| **Biofeedback** | **Woodford 2007** (EMG biofeedback)  **Molier 2010** (qualitative data only) |  |  |  | Current evidence of low quality | Insufficient evidence to support any change in current clinical practice | Up-to-date reviews required |
| **Bobath therapy** | **Luke 2004** |  |  |  | Current evidence of low quality | Insufficient evidence to support any change in current clinical practice | Up-to-date reviews required |
| **Brain stimulation: tDCS** | **Elsner 2013** |  | **tDCS beneficial** for impairment (7 trials, n = 304) | High-quality evidence of **no benefit or harm of tDCS** (5 trials, n = 286) |  | Evidence insufficient to support introduction into routine clinical practice | High-quality RCTs required |
| **Brain stimulation: rTMS** | **Hao 2013** |  |  |  | Current evidence of low quality | Insufficient evidence to support introduction into routine clinical practice | High-quality RCTs required |
| **Constraint-induced movement therapy (CIMT)** | **Corbetta 2010** (subgroup analyses)  **Sirtori 2009** | **CIMT beneficial** when compared with control (14 trials, n = 477) |  |  | Evidence of low quality for measures of ADLs (because of methodological limitations within review) | Moderate-quality evidence that CIMT may be effective intervention for selected patients | Phase III RCTs recommended Dose must be considered |
| **Electrical stimulation** | **Farmer 2014**  **Nascimento 2014**  **Meilink 2008** |  |  |  | Current evidence of low quality | Insufficient evidence to support any change in current clinical practice | Meta-analysis of current trials/completion of ongoing review required (Howlett) |
| **"Hands-on" therapy (manual therapy techniques)** | **Winter 2011** (qualitative data only) |  |  |  | Current evidence of low quality | Insufficient evidence to support any change in current clinical practice | High-quality RCTs required |
| **Mental practice** | **Barclay-Goddard 2011** (subgroup analyses)  **Braun 2013**  **Wang 2011** (includes Chinese trials) | **Mental practice beneficial** when given in addition to conventional interventions (7 trials, n = 197) | **Mental practice beneficial** when given in addition to conventional interventions (5 trials, n = 216) | **No benefit or harm** of mental practice |  | Moderate-quality evidence that mental practice may be effective intervention for some patients | Phase III RCTs recommended |
| **Mirror therapy** | **Thieme 2012** | **Mirror therapy beneficial** (10 trials, n = 421): combined upper limb function and impairment outcomes | (see upper limb function) | **Mirror therapy beneficial** (4 trials, n = 217) |  | Moderate-quality evidence that mirror therapy may be effective intervention for some patients | Phase III RCTs recommended |
| **Music therapy** | **Bradt 2010** |  |  |  | Lack of trial evidence | Insufficient evidence to support any change in current clinical practice | High-quality RCTs required |
| **Pharmacological interventions** | **Elia 2009** (botulinum toxin for spasticity)  **Olvey 2010** (botulinum toxin for spasticity; qualitative data only)  **Demetrios 2013** (multi-disciplinary rehabilitation following pharmacological interventions; qualitative data only)  **Singh 2010** (pharmacological interventions for shoulder pain) |  |  |  | Current evidence of low quality | Insufficient evidence to support any change in current clinical practice | Reviews require updating  High-quality RCTs required |
| **Repetitive task training (RTT)** | **French 2007**  **French 2008** | **No benefit or harm** of RTT (8 trials, n = 412)  **Beneficial effect when dose > 20** hours (3 trials, n = 113) |  |  |  | Moderate-quality evidence that a higher dose of RTT  may be beneficial | Review requires updating  Large-scale RCTs to explore dose is a research priority, including number of repetitions during RTT |
| **Robotics** | **Mehrholz 2012**  **Norouzi-Gheidari 2012** |  | **Beneficial effect of robotics as compared with any comparison** on impairment scales (16 trials, n = 586)  **No benefit or harm as compared with the same duration** of conventional therapy (6 trials, n = 204)  No benefit or harm on measures of strength (10 trials, n = 321) | **Beneficial effect** of robotics as compared with any comparison on ADLs (13 trial, n = 552) |  | Current evidence does not support Introduction into routine clinical practice | High-quality RCTs required, including consideration of dose |
| **Sensory interventions** | **Doyle 2010**  **Schabrun 2009**(qualitative data only) | **Beneficial effect** of sensory stimulation as compared with no treatment (1 trial, n = 29) | **Beneficial effect** of sensory stimulation as compared with no treatment (1 trial, n = 29) |  | Low-quality evidence for all other interventions | Current evidence does not support any change in current clinical practice | High-quality RCTs required |
| **Strength training** | **Harris 2010** |  |  |  | **Low-quality evidence of a beneficial effect** on upper limb function (11 trials, n = 465) and grip strength (6 trials, n = 306). (Quality judgement influenced by poor reporting within review) | Insufficient evidence to support any change in current clinical practice | High-quality up-to-date review required  High-quality RCTs required |
| **Stretching and positioning** | **Katalinic 2010** (stretching and positioning)  **Borisova 2009** (positioning of shoulder)  **Ada 2005** (shoulder supports)  **Lannin 2003** (hand splinting)  **Hijmans 2004** (elbow orthoses; qualitative data only) |  | **No benefit or harm** of stretching as compared with any other intervention on joint mobility and spasticity | **No benefit or harm** of stretching as compared with any other intervention on ADLs | **Low-quality evidence of no benefit** of shoulder supports | Current evidence does not support any change in current clinical practice | High-quality up-to-date review required  Essential that research protocols comprise doses that are theoretically predicted to effect change |
| **Task-specific training (reach-to-grasp exercise)** | **Pelton 2012** (qualitative data only)  **Urton 2007** (qualitative data only) |  |  |  | Current evidence of low quality | Insufficient evidence to support any change in current clinical practice | High-quality, up-to-date review required |
| **Virtual reality** | **Laver 2011** | **Virtual reality beneficial** (7 trials, n = 205): combined upper limb function and impairment outcomes | (see upper limb function)  No benefit or harm for grip strength (2 trials, n = 44) |  |  | Moderate-quality evidence that virtual reality may be effective intervention for some patients | Phase III RCTs recommended, including consideration of dose |

KEY: ADLs: Activities of daily living. EMG: Electromyography. RCTs: Randomised controlled trials. rTMS: Repetitive transcranial magnetic stimulation. tDCS: Transcranial direct current stimulation
