# Supplementary material for: Selecting and implementing overview methods: implications from five exemplar overviews
Source: Syst Rev. 2017 Jul 18;6:145. doi: 10.1186/s13643-017-0534-3 (PMC5516331; doi:10.1186/s13643-017-0534-3)
Supplement: Supplementary file 2 — Summary of findings as presented by Brunton [12]. (DOCX 20 kb) [file 13643_2017_534_MOESM2_ESM.docx]

**Additional File 2: Summary of results tables proposed by McClurg 2016 [10] and Escourt 2016 [9]:**

McClurg 2016 [10] Draft summary of results table. Separate summary of results tables are planned for each of the stated primary and secondary outcomes of interest to this overview.

(Table adapted from [37]).

|  | **Important difference** | **Small difference (may not be important)** | **Little or no difference** |
| --- | --- | --- | --- |
| **High certainty evidence** | INTERVENTIONS (insert list) which improve/decrease/prevents OUTCOME | INTERVENTIONS (insert list) which improves slightly/decreases slightly OUTCOME | INTERVENTIONS (insert list) which results in little or no difference in OUTCOME |
| **Moderate certainty evidence** | INTERVENTIONS (insert list) which probably improve/decrease/prevents OUTCOME | INTERVENTIONS (insert list) which probably improves slightly/decreases slightly OUTCOME | INTERVENTIONS (insert list) which probably results in little or no difference in OUTCOME |
| **Low certainty evidence** | INTERVENTIONS (insert list) which may improve/decrease/prevents OUTCOME | INTERVENTIONS (insert list) which may improve slightly/decrease slightly OUTCOME | INTERVENTIONS (insert list) which may result in little or no difference in OUTCOME |
| **Very low certainty evidence** | It is uncertain whether INTERVENTIONS (insert list) improves/decreases/prevents OUTCOME because the certainty of the evidence is low | | |
| **No data or no studies** | OUTCOME was not measured or not reported or no studies were found that evaluated the impact of INTERVENTION on OUTCOME | | |

**Estcourt 2016 [9]. Cochrane Handbook - Figure 22.3.b: Template for an ‘Overview of reviews’ table [4]**

| **Interventions for [Condition] in [Population]** | | | | | | | |
| --- | --- | --- | --- | --- | --- | --- | --- |
| **Outcome** | **Intervention and Comparison intervention** | Illustrative comparative risks (95% CI) | | **Relative effect (95% CI)** | **Number of participants (studies)** | **Quality of the evidence (GRADE)** | **Comments** |
|  |  | Assumed risk | Corresponding risk |  |  |  |  |
|  |  | **With comparator** | **With intervention** |  |  |  |  |
| Outcome #1 | | | | | | | |
|  | Intervention/Comparison #1 |  |  |  |  |  |  |
|  | Intervention/Comparison #2 |  |  |  |  |  |  |
|  | Etc… |  |  |  |  |  |  |
| Outcome #2 | | | | | | | |
|  | Intervention/Comparison #1 |  |  |  |  |  |  |
|  | Intervention/Comparison #2 |  |  |  |  |  |  |
|  | Etc… |  |  |  |  |  |  |
| Outcome #3 | | | | | | | |
|  | Intervention/Comparison #1 |  |  |  |  |  |  |
|  | Intervention/Comparison #2 |  |  |  |  |  |  |
|  | Etc… |  |  |  |  |  |  |
