# Supplementary material for: Selecting and implementing overview methods: implications from five exemplar overviews
Source: Syst Rev. 2017 Jul 18;6:145. doi: 10.1186/s13643-017-0534-3 (PMC5516331; doi:10.1186/s13643-017-0534-3)
Supplement: Supplementary file 3 — Summary of results tables proposed by McClurg [10] and Escourt [9]. (DOCX 22 kb) [file 13643_2017_534_MOESM3_ESM.docx]

**Additional File 3. Summary of findings as presented by Brunton 2016** [12]

From Brunton 2016, Appendix 8: Direction of effect for health and business outcomes

**Table A8.1:** Direction of effect for all health and business outcomes

| **Review** | **AMSTAR rating** | **++** | **+** | **0** | **-** | **--** |
| --- | --- | --- | --- | --- | --- | --- |
| Abraham and Graham-Rowe (2009) | 4 | Physical activity; Physical fitness |  |  |  |  |
| Anderson et al. (2009) | 10 | BMI; Weight |  |  |  |  |
| Aniol (2001) | 5 | **Absenteeism;** Smoking cessation |  |  |  |  |
| Archer et al. (2011) | 10 | Weight |  |  |  |  |
| Baicker et al. (2010) | 4 |  | Absenteeism costs; Saving per employee |  |  |  |
| Cahill et al. (2008) | 9 | Smoking cessation | Smoking cessation |  |  |  |
| Conn et al. (2009) | 7 | **Absenteeism;** Anthropometric measures; Diabetes risk; Fitness; Healthcare utilisation; **Job Stress;** Lipids | Mood; Quality of life |  |  |  |
| Dishman et al. (1998) | 5 |  | Physical activity |  |  |  |
| Hutchinson and Wilson (2012) | 4 | Activity; Cholesterol; Diastolic BP; Fat intake; Fitness; Fruit intake; Vegetable intake; Weight |  | Cholesterol |  | Weight |
| Kremers et al. (2010) | 7 | Weight |  |  |  |  |
| Kuoppala et al. (2008) | 7 | Work ability | **Absenteeism;** Mental wellbeing; Physical wellbeing; Wellbeing; **Work ability** |  |  |  |
| Leeks et al. (2010) | 9 |  | Smoking cessation |  |  |  |
| Martin et al. (2009) | 8 | Anxiety; Depression | Mental health |  |  |  |
| McLeod (2010) | 6 |  | Psychological outcomes |  |  |  |
| Montano et al. (2014b) | 9 | BMI; **Job stress;** Musculoskeletal symptoms; Fruit and vegetable intake |  |  |  |  |
| Ojo et al. (2011) |  | Self-reported STD; Sex with a commercial sex worker; Unprotected sex; Voluntary counselling and testing | Alcohol before sex; Multiple sexual partners |  | HIV incidence |  |
| Parks and Steelman (2008) | 10 | Absenteeism; Job satisfaction |  |  |  |  |
| Richardson and Rothstein (2008) | 6 | Psychological outcome |  |  |  |  |
| Rongen et al. (2013) | 10 | Absenteeism; Health; Productivity | Work ability |  |  |  |
| Smedslund et al. (2004) | 9 | Smoking cessation |  |  |  |  |
| Tan et al. (2014) | 10 | Depression |  |  |  |  |
| Thomson and Ravia (2001) | 9 |  | Fruit and vegetable intake |  |  |  |
| van Dongen et al. (2011) | 9 | Absenteeism costs; Medical costs |  |  |  | Absenteeism costs; Medical costs |
| Verweij et al. (2011) | 10 | BMI, Body fat percentage; Weight | Body fat percentage |  |  |  |
| ++ statistically significant beneficial effect; + non-significant beneficial effect; 0 no difference between control and intervention; - non-significant detrimental effect; -- statistically significant detrimental effect; business outcomes in bold. | | | | | | |

**Table A8.2:** Direction of effect for different intervention types

| **Review** | **AMSTAR rating** | **++** | **+** | **0** | **-** | **--** |
| --- | --- | --- | --- | --- | --- | --- |
| Abraham and Graham-Rowe (2009) | 4 | Exercise |  |  |  |  |
| Aniol (2001) | 5 | Mental health interventions |  |  |  |  |
| Anderson et al. (2009) | 10 | Nutrition and/or PA |  |  |  |  |
| Archer et al. (2011) | 10 | PA; Education; Access to healthy food; Environmental change; Exercise prescription; Competitions and incentives; Multicomponent |  |  |  |  |
| Baicker et al. (2010) | 4 |  | Health risk assessment; Self-help education materials; Individual counselling; Classes, seminars, group activities; Added incentives for participation |  |  |  |
| Cahill et al. (2008) | 9 | Group therapy; Individual counselling; Pharmacological interventions; Incentives; Multicomponent | Self-help interventions |  |  |  |
| Conn et al. (2009) | 7 | PA interventions | PA interventions |  |  |  |
| Dishman et al. (1998) | 5 |  | Exercise |  |  |  |
| Hutchinson and Wilson (2012) | 4 | Education; Cognitive-behavioural; Exercise; Motivation enhancement; Social influence |  | Education |  | Education; Cognitive-behavioural; Motivation enhancement |
| Kremers et al. (2010) | 7 | Diet and PA; Diet alone; PA alone |  |  |  |  |
| Kuoppala et al. (2008) | 7 | Education; Ergonomics; PA; Lifestyle; Psychological; Work redesign | Education; Ergonomics; PA; Lifestyle; Psychological; Work redesign |  |  |  |
| Leeks et al. (2010) | 9 |  | Incentives and competitions |  |  |  |
| Martin et al. (2009) | 8 | Psychoeducation focused on cognitive behaviour or training in coping skills for stress management; PA | Psychoeducation focused on cognitive behaviour or training in coping skills for stress management; PA |  |  |  |
| McLeod (2010) | 6 |  | Counselling |  |  |  |
| Montano et al. (2014b) | 9 | Cognitive behavioural; Ergonomics; Education; PA; Stress management; Multicomponent |  |  |  |  |
| Ojo et al. (2011) |  | Advice, Education; Voluntary counselling and testing; Peer education | Voluntary counselling and testing; Education |  | Voluntary counselling and testing |  |
| Parks and Steelman (2008) | 10 | Education; Exercise; Multicomponent |  |  |  |  |
| Richardson and Rothstein (2008) | 6 | Cognitive-behavioural; Multi-component; Relaxation; Miscellaneous (exercise, EMG feedback, journalling, skills development, classroom management training for teachers) | Social support |  |  |  |
| Rongen et al. (2013) | 10 | Advice; Counselling; Education; Exercise; Incentives; Stress management; Multicomponent | Advice; Counselling; Education; Exercise; Incentives; Stress management; Multicomponent |  |  |  |
| Smedslund et al. (2004) | 9 | Self-help manuals; Physician advice, Health education, Cessation groups, Incentives; Competitions |  |  |  |  |
| Tan et al. (2014) | 10 | Cognitive behavioural therapy; Education; Exercise; Skill development; Social support | Cognitive behavioural therapy |  |  |  |
| Thomson and Ravia (2001) | 9 |  | Counselling; Diet feedback; Education; Environmental change; Peer mentoring |  |  |  |
| Van Dongen et al. (2011) | 9 | Nutrition and/or physical activity; Multicomponent |  |  |  | Nutrition and/or PA; Multicomponent |
| Verweij et al. (2011) | 10 | PA and dietary behaviour interventions (Advice; Education; Feedback; Incentives; PA; Screening; Counselling; Multicomponent) | PA and dietary behaviour interventions (Advice; Education; Feedback; Incentives; PA; Screening; Counselling; Multicomponent) |  |  |  |
| ++ statistically significant beneficial effect; + non-significant beneficial effect; 0 no difference between control and intervention; - non-significant detrimental effect; -- statistically significant detrimental effect; PA physical activity | | | | | | |
| From Brunton G, Dickson K, Khatwa M, Caird J, Oliver S, Hinds K, Thomas J (2016) *Developing evidence-informed, employer-led workplace health.* London: EPPI-Centre, Social Science Research Unit, Institute of Education, University College London. | | | | | | |
